# Antibody dynamics in children with first or repeat *Plasmodium falciparum* infections

**DOI:** 10.3389/fmed.2022.869028

**Published:** 2022-07-19

**Authors:** Eric Rogier, Doug Nace, Pedro R. Dimbu, Brian Wakeman, James G. Beeson, Chris Drakeley, Kevin Tetteh, Mateusz Plucinski

**Affiliations:** ^1^Malaria Branch, Division of Parasitic Diseases and Malaria, Centers for Disease Control and Prevention, Atlanta, GA, United States; ^2^National Malaria Control Program, Luanda, Angola; ^3^Burnet Institute, Melbourne, VIC, Australia; ^4^Central Clinical School, Monash University, Melbourne, VIC, Australia; ^5^Department of Medicine, University of Melbourne, Melbourne, VIC, Australia; ^6^London School of Hygiene & Tropical Medicine, London, United Kingdom; ^7^U.S. President’s Malaria Initiative, Centers for Disease Control and Prevention, Atlanta, GA, United States

**Keywords:** malaria, antibodies, isotypes, boosting, exposure

## Abstract

Immunoglobulin (Ig) production during and after infection with *Plasmodium* parasites is one of the greatest adaptive immune defenses the human host has against this parasite. Infection with *P. falciparum* has been shown to induce different B cell maturation responses dependent upon the age of the patient, number of previous exposures, and severity of the disease. Described here are dynamics of Ig responses to a panel of 32 *P. falciparum* antigens by patients followed for 42 days and classified individuals as showing characteristics of an apparent first *P. falciparum* infection (naïve) or a repeat exposure (non-naïve). Six parameters were modeled to characterize the dynamics of IgM, IgG_1_, IgG_3_, and IgA for these two exposure groups with differences assessed among Ig isotypes/subclasses and unique antigens. Naïve patients had significantly longer periods of time to reach peak Ig titer (range 4–7 days longer) and lower maximum Ig titers when compared with non-naïve patients. Modeled time to seronegativity was significantly higher in non-naïve patients for IgM and IgA, but not for the two IgG subclasses. IgG_1_ responses to Rh2030, HSP40, and PfAMA1 were at the highest levels for non-naïve participants and may be used to predict previous or nascent exposure by themselves. The analyses presented here demonstrate the differences in the development of the Ig response to *P. falciparum* if the infection represents a boosting response or a primary exposure. Consistency in Ig isotype/subclasses estimates and specific data for *P. falciparum* antigens can better guide interpretation of seroepidemiological data among symptomatic persons.

## Introduction

The human host mounts a vigorous adaptive immune response to the *Plasmodium falciparum* parasite, and B cell responses through antibody-mediated immunity have been shown to be protective against malaria (1), and even passive transfer of serum antibodies from persons living in endemic settings reduces *P. falciparum* parasite burden of symptomatic children (2). As malaria infection is a bloodborne infectious disease, anti-*P. falciparum* immunoglobulin (Ig) titers are generally highest for IgG in humans upon natural exposure, though both IgM and IgA antibodies are observed in substantial quantities as well (3–5). The IgG response to *P. falciparum* can be further subdivided by the four subclasses of this isotype in humans, with highest serum levels of IgG_1_ followed by IgG_3_, IgG_4_, and IgG_2_ (6–9); however, the relative abundance of IgG subclass response varies for different malaria antigens. IgG1 and IgG3 are generally the predominant response and effectively mediate interactions with complement and Fcγ-receptors expressed on immune cells, which play roles in immunity (10, 11). Understanding the induction, function, and dynamics of the Ig response against *P. falciparum* antigens has greatly enhanced vaccine development (12–14) and interpretation of seroepidemiological studies (15, 16).

In *P. falciparum-*endemic areas throughout the world, infants or children may become exposed to this parasite at a very early age. Passive placental transfer of IgG to the fetus provides a degree of clinical protection early in life (17), though these antibodies are generally lost by 6 months of age (18, 19). Formation and maturation of the host anti-*P. falciparum* B cell response is an area of active research for several decades, though human studies have obvious limitations due to the inherent need for immediate treatment when any infection is diagnosed. The response in humans appears to follow many of the classical assumptions regarding class switching, affinity maturation, and clonal selection, though recent work has emphasized the contribution of atypical B cell populations early in development which appear be able to respond faster to antigen challenge, but are less efficient at establishing protective and long-term antibody production (20–22). Evidence has been presented showing the early B activation in response to *P. falciparum* in naïve humans to be dominated by short-lived and metabolically-active plasmablasts which have the capacity for prolific antibody secretion, but may inhibit the formation of durable immunity (23). Additionally, as with B cell maturation to many other immunogenic agents, the importance of CD4 + T follicular helper cells has been documented for the development of the *P. falciparum* antibody response with T helper (Th) cell Th1 and Th2 subsets likely playing distinct roles (24, 25). Recent studies have also highlighted the prominence of IgM responses to malaria infection, including repeat infections and the persistence of IgM responses over time (26, 27).

Individual *P. falciparum* antigens have been identified for their specific abilities to induce B cell responses and antibody production in exposed endemic populations (16, 28–31), as well as controlled human malaria infections (CHMIs) (24, 26, 32, 33). The study presented here investigates the short-term immunoglobulin response to natural *P. falciparum* infection by categorizing a study population of children into first or repeat infection and comparing Ig responses for 42 days following antimalarial treatment. This study aims to understand the Ig dynamics arising from a B cell response in the nascent host vs. a host with previous immunological memory. Ig responses were broadly investigated in a population of children against a panel of 32 *P. falciparum* antigens encompassing all life stages in the human host was investigated for the ability to bind IgM, IgG_1_, IgG_3_, and IgA in blood samples in order to obtain detail on the specificity and nature of immune responses. These data are used to estimate the nascent B cell response to *P. falciparum* exposure through the dynamics of Ig production to multiple parasite antigens, and how this differs from individuals experiencing a repeat *P. falciparum* infection.

## Materials and methods

### Study design

Dried blood spots were collected during a therapeutic efficacy monitoring study (TES) in Angola in 2017 (34). Samples from all three sentinel TES sites were included: high-transmission M’Banza Congo, Zaire Province and Saurimo, Lunda Sul Province; and low\mid-transmission Benguela, Benguela Province. Due to different transmission levels, the parasite density criteria for enrollment were lower in Benguela province (1,000–100,000 p/μL blood) vs. Lunda Sul and Zaire (2,000–200,000 p/μL blood). Children aged 6 months to 11 years old with microscopically confirmed acute *P. falciparum* infection were treated with one of three artemisinin-based combination therapies (ACT) and followed weekly for 28 (participants treated with artemether-lumefantrine or artesunate-amodiaquine) or 42 (participants treated with dihydroartemisinin-piperaquine) days. Patients with severe or complicated malaria infections were excluded from enrollment. Participant samples were collected on Days 0 (enrollment), 2, 3, 7, 14, 21, 28, 35, and 42 after initiation of ACT.

### Ethics approval

Study participants consented to collection of malaria data from provided blood samples. The study received human subjects approval from the Angolan Ministry of Health. Secondary analysis of anonymized samples was approved by the office of the Associate Director of Science in the Center for Global Health at the CDC (Project ID: 0900f3eb8193aa9d).

### Laboratory analysis

Samples were assayed for antibody responses to a panel of *P. falciparum* antigens using a multiple bead-based assay as described previously (5). Assay signal was provided as mean fluorescent intensity (MFI) minus the signal from blank wells on each plate to provide a final signal of MFI-bg for analysis.

### Statistical analysis

To classify study participants into *P. falciparum* naïve (first lifetime infection) and non-naïve (repeat infection) categories, the assay signals for baseline (Day 0) samples for IgG_1_ response to PfMSP1 and PfAMA1 were compared. Natural exposure to PfMSP1 and PfAMA1 is highly immunogenic in humans, and IgG_1_ responses to these two antigens are long-lived and generally considered to be indicative of any prior exposure to *P. falciparum* (5, 35–37). MFI-bg seropositivity thresholds for PfMSP1 and PfAMA1 were 115 and 113, respectively, and generated as described previously (5). Children that had IgG_1_ responses to both the PfMSP1 and PfAMA1 antigens below the seropositivity threshold at Day 0 were considered “naive” and it was assumed that their presenting *P. falciparum* infection was their first-ever *P. falciparum* infection. This classification scheme is also supported by the three TES enrollment sites being located in meso- to high-endemic *P. falciparum* settings in Angola (34), and the relatively young ages of participants, meaning it is unlikely they would have had time for IgG_1_ seroreversion from a previous *P. falciparum* exposure. In order to have higher confidence in the classification scheme, children seropositive for PfMSP1 and/or PfAMA1 IgG_1_ but having assay signal less than one log_10_ fold greater than the seropositivity threshold (MFI-bg value of 1,150 and 1,130, respectively) were classified as indeterminate and excluded from further analysis. All other children with high IgG_1_ assay signals to these *P. falciparum* antigens were considered “non-naive” with strong evidence for previous *P. falciparum* blood-stage infection.

For all antigens included in the panel, individual decay curves were characterized with six key parameters: C_max_, the maximum antibody signal; Δ_C_, the difference between C_max_ and the antibody signal at Day 0; C_end_, the antibody signal at last day of follow up; t_max_, the time in days to maximum antibody signal; t_1/2_, the post-peak half-life; and t_neg_, the expected time to seronegativity (5). The distribution of the estimates for these parameters across all antigens was compared between children classified as naive and non-naive. The analysis was separately done for IgG_1_, IgG_3_, IgM, and IgA. Though IgG2, IgG4, IgE, and IgD responses were also measured in these same samples, these isotypes/subclasses were either undetectable (at 1:100 serum concentration) or too few children displayed responses for these Igs to allow for parameter estimates (5).

Differences in the empirical distributions were assessed using the Kolmogorov–Smirnov non-parametric test. Heatmaps were generated to simultaneously assess clustering patterns of antibody responses by antigen and participant. All analysis was done in R version 3.6.0 (R Foundation for Statistical Computing, Vienna, Austria).

## Results

### Classification of study population into *Plasmodium falciparum* naïve and non-naïve

**FIGURE 1:**
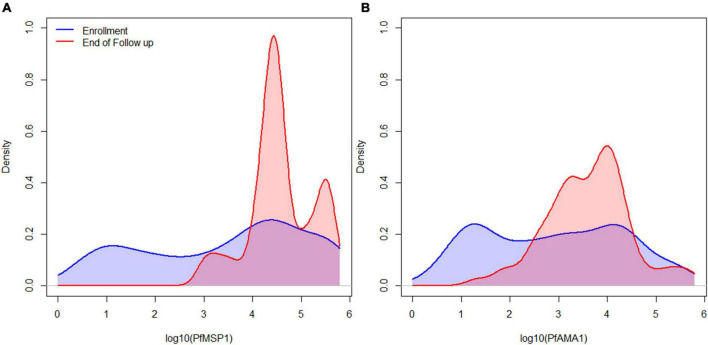
IgG_1_ antibody levels for all participants for the immunogenic *P. falciparum* PfMSP1 and PfAMA1 antigens. Smoothed distribution of IgG_1_ antibody responses to PfMSP1 **(A)** and PfAMA1 **(B)** at enrollment (baseline) in blue, and last day of follow-up in red for children treated for *P. falciparum* infection.

**FIGURE 2:**
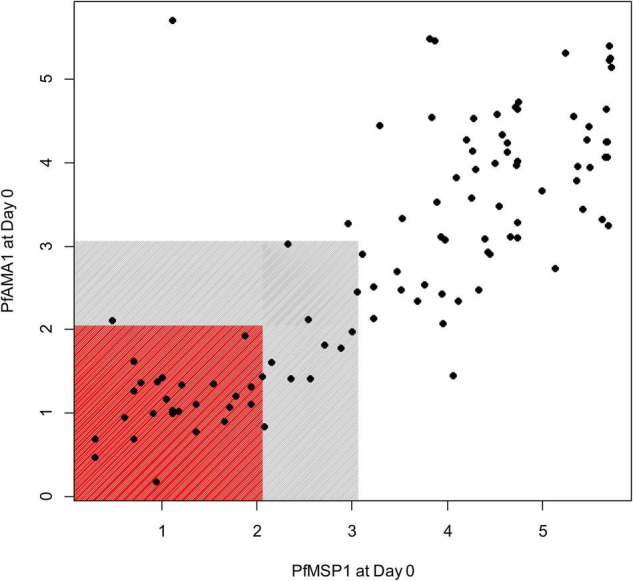
Relationship of the IgG_1_ antibody responses to PfMSP1 and PfAMA1 at the first day of follow-up (Day 0) in children treated for *P. falciparum* infection. Axes display assay log_10_-transformed assay signal to the two respective *P. falciparum* antigens. Children IgG_1_ seronegative for both antigens are shown by markers in the red box, and children nominally IgG_1_ seropositive to either (or both) antigens shown in gray box. Children seronegative to both targets are classified as *P. falciparum* naïve, and children with assay signals greater than the gray shading classified as non-naïve.

Of 104 enrolled participants, 89 (85.6%) provided samples for the entire 42-day follow-up period (Days 0, 2, 3, 7, 14, 21, 28, 35, and 42 after enrollment), while 2 (1.9%) provided samples only up to 35 days, 1 (1.0%) to 28 days, 6 (5.8%) to 21 days, and 6 (5.8%) to 14 days. As described and listed previously, a total of 32 *P. falciparum* antigens were utilized for the multiplex antibody detection assay (5). The distribution of IgG_1_ responses to PfMSP1 and PfAMA1 for the entire study population were bimodal at Day 0, and by the end of follow-up sampling, nearly all participants registered high responses to both of these antigens (Figure 1). A similar pattern was observed for the IgG_3_ response to both antigens, but the distributions were not as distinct for IgM and IgA antibodies (Supplementary Figure 1). As described in “Materials and methods,” the bivariate correlation for participant IgG_1_ responses to PfMSP1 and PfAMA1 at day of enrollment (Day 0) was considered in order to classify participants as previously exposed to *P. falciparum* (participant was non-naïve), or if the current infection represented potential first *P. falciparum* exposure (participant was naïve). Using the PfMSP1 and PfAMA1 assay signal thresholds for IgG_1_, of the 104 participants, 26 (25%) were classified as naïve (seronegative to both antigens), 66 (63%) as non-naïve (high levels of IgG_1_ to either), and 12 as indeterminate (seropositive to either, but of lower assay signal) (Figure 2). Of the 66 persons classified as non-naive, 64 (97.0%) were seropositive to both of these antigens. For the remaining two individuals, one was IgG_1_ seropositive to PfMSP1 only, and one was seropositive to PfAMA1 only. Participants classified as exposure naïve were on average 1.2 years younger than non-naive participants (mean age 2.3 years vs. 3.5, Student’s *t*-test *p*-value 0.001). Parasite density at Day 0 and sex were not statistically different between the two classification groups (Table 1).

**TABLE 1:** Characteristics of children with *P. falciparum* infection stratified into exposure naïve and exposure non-naïve.

	Non-naïve (*n* = 66)	Naïve (*n* = 26)	*P*-value*
Age, mean (standard deviation)	3.5 (± 1.9)	2.3 (± 1.3)	0.001
Parasite density at Day 0 (pg/μL), median (range)	23704 (3916–184465)	15682 (4113–184243)	0.500
Female, %	48%	50%	1.000

*Difference in age was assessed using a t-test, difference in parasitemia using a t-test after log-transformation, and difference in sex using a chi-square test.

### Differences in parameter estimates by Ig isotype and subclass

**FIGURE 3:**
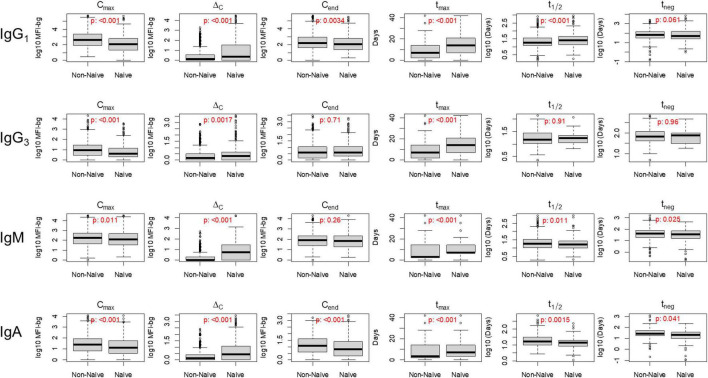
Distribution of six key post-treatment clearance parameters in non-naïve and naïve Angolan children treated for malaria, stratifying by immunoglobulin (Ig) class/subclass. *P*-value for Kolmogorov–Smirnov test for difference in empiric distribution between non-naïve and naïve participants. Mean values between non-naïve and naïve categories are displayed in Table 2.

When stratifying by Ig isotype and subclass for IgG_1_, IgG_3_, IgM, and IgA, considerable differences were observed in many of the six parameters used to describe Ig dynamics between the naïve and non-naïve groups for the aggregate responses to the 32 *P. falciparum* antigens (Figure 3). For IgG_1_, five of the six parameters showed statistically significant differences between the naïve and non-naïve groups (Table 2). Of particular note was the difference in IgG_1_ C_max_ (MFI-bg of 407 for non-naïve vs. 117 for naïve) and in t_max_, which was 7 days later for the naïve IgG_1_ response. Aggregate estimates for three of the five parameters for dynamics in IgG_3_ response were significantly different with C_max_ again higher for non-naives, and t_max_ again 1 week later for naives. For IgM, five of the six parameters showed significant differences in aggregate estimates, and for IgA, all six parameters were significantly different between the two groups. When considering the six parameter estimates for all malaria antigens and Ig isotypes together, naïve participants had significantly lower maximum antibody responses (C_max_), higher absolute changes in antibody response (Δ_C_), took longer to reach maximum antibody response (t_max_), and were seropositive for a shorter period of time (t_neg_) compared to non-naive participants (*p*-values < 0.001) (Supplementary Figure 2).

**TABLE 2:** Mean values for each parameter by immunoglobulin isotype/subclass as aggregate for all *P. falciparum* antigen responses estimated for the naïve and non-naïve children.

		C_max_ (log_10_ MFI-bg)	Δ_C_ (log_10_ MFI-bg)	C_end_ (log_10_ MFI-bg)	t_max_ (days)	t_1/2_ (days)	t_neg_ (days)
IgG_1_	Non-Naïve	**2.61**	**0.12**	**2.23**	**7.0**	**19.5**	66.1
	Naïve	**2.07**	**0.34**	**2.04**	**14.0**	**24.5**	53.7
	*p*-value	**<0.001**	**<0.001**	**0.003**	**<0.001**	**<0.001**	0.06
IgG_3_	Non-Naïve	**0.95**	**0.19**	0.60	**7.0**	14.8	64.6
	Naïve	**0.60**	**0.30**	0.60	**14.0**	17.0	75.9
	*p*-value	**<0.001**	**0.002**	0.71	**<0.001**	0.91	0.96
IgM	Non-Naïve	**2.23**	**0.00**	1.89	**3.0**	**18.6**	**39.8**
	Naïve	**2.09**	**0.74**	1.83	**7.0**	**16.6**	**33.9**
	*p*-value	**0.011**	**<0.001**	0.26	**<0.001**	**0.011**	**0.025**
IgA	Non-Naïve	**1.38**	**0.11**	**1.04**	**3.0**	**17.0**	**28.8**
	Naïve	**1.11**	**0.40**	**0.81**	**7.0**	**13.5**	**21.4**
	*p*-value	**<0.001**	**<0.001**	**<0.001**	**<0.001**	**<0.002**	**0.041**

*Statistically significant mean differences are displayed in bold.*

### Differences in specific *Plasmodium falciparum* antigen Ig responses by participant classification

**FIGURE 4:**
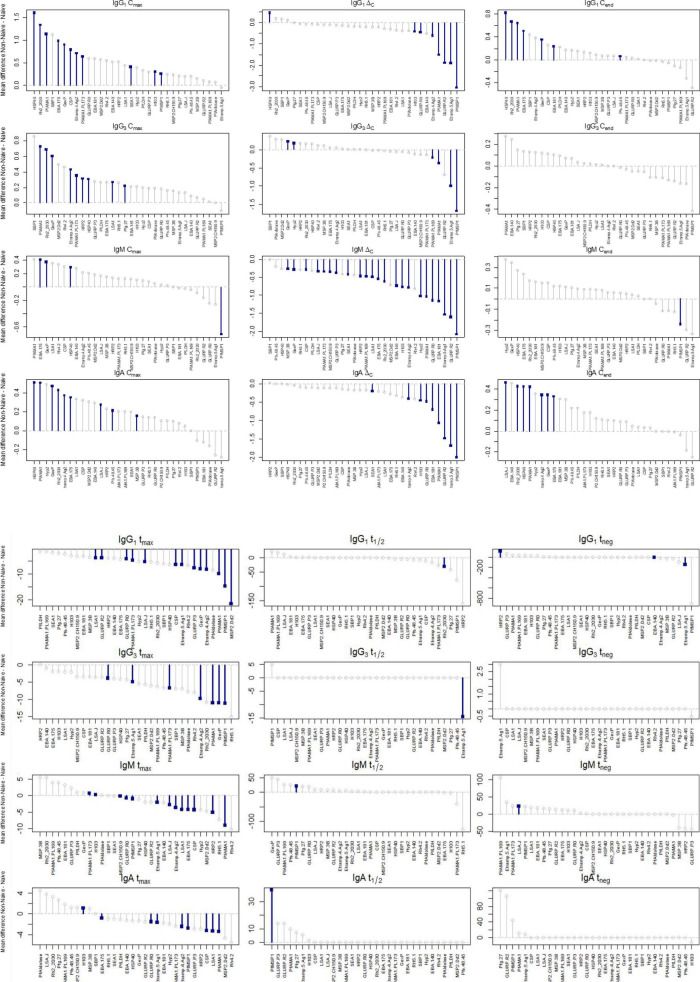
Mean differences for each parameter by Ig response for non-naïve and naïve children followed post-treatment. Positive values represent higher values in non-naïves, and lines with blue squares denote statistically significant differences.

When assessing differences in Ig dynamics parameter estimates for individual *P. falciparum* antigens, some general trends were observed, but with variability within each isotype/subclass. Certain antigens were particularly discriminatory between naive and non-naive participants. For example, HSP40, Rh2030 and PfAMA1 tended to have a substantially higher C_max_ and C_end_ in non-naive than naive participants for the IgG_1_ response (Figure 4 and Supplementary Figure 3). All three of these antigens also stimulated significantly higher IgA responses on the last day of follow-up in non-naive vs. naive participants. Interestingly, C_max_ for the anti-PfMSP1 IgM response was substantially higher in naïve participants, which translated to significantly higher Δ_C_ and C_end_ estimates in naïve persons. Estimates for t_max_, t_1/2_, and t_neg_ yielded some striking findings for individual antigens: t_max_ 21 days longer for MSP2_Dd2 for IgG_1_ in naïve persons, t_1/2_ 30 days longer for Rh_2030 for IgG_1_ in naïve persons, t_1/2_ 15 days longer for Etramp5 Ag1 for IgG_3_ in naïve persons, and t_1/2_ significantly longer for IgM (25 days) and IgA (39 days) against PfMSP1 in non-naïve persons. In time to apex antibody levels (t_max_), all isotypes showed the predominance of naïve children taking longer to reach this apex with statistically significant differences for 13/32 (40.6%) of antigens for IgG_1_ detection, 7/32 (21.9%) of antigens for IgG_3_, 11/32 (34.4%) for IgM, and 8/32 antigens (25.0%) for IgA. Antibodies against the PfMSP1, PfAMA1, GLURP R_o_, Etramp4Ag2, and Etramp5Ag1 antigens were all significantly delayed in reaching apex levels in naïve children for all four Ig isotypes/sub-classes.

### Hierarchical clustering based on Ig response parameters

**FIGURE 5:**
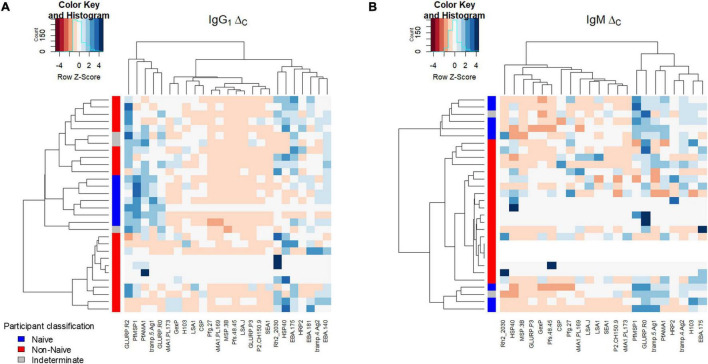
Hierarchical relationship among study participants shown for the Δ_C_ parameter. Clustering of Δ_C_ by *P. falciparum* antigen response (columns) and participants (rows) for IgG_1_ **(A)** and IgM **(B)**. Intensity of color shading within the plot indicated by the magnitude of the standard score (Z score).

Estimates among the panel of *P. falciparum* antigens for Δ_C_ for IgG_1_ and IgM were sufficiently consistent to allow clustering based on naïve or non-naïve classification (Figure 5). Clustering based on IgG_1_ Δ_C_ was largely driven by higher estimates in the naïves for PfMSP1, PfAMA1, and Etramp5Ag1, and lower estimates in Rh_2030, HSP40, HRP2, Etramp4Ag2, and the three EBA antigens. Clustering based on IgM Δ_C_ was largely driven by the higher estimates in naïves for PfMSP1, GLURP R_o_, Etramp5Ag1, and PfAMA1. For children classified as indeterminate (neither naïve or non-naïve), the IgG_1_ Δ_C_ estimates among antigens did not cluster with either naïve/non-naïve categories, though the IgM Δ_C_ estimates were more similar with the children classified as naïve.

## Discussion

Here is described the quantitative comparison of six parameters describing Ig dynamics upon successful treatment of *P. falciparum* infection when study participants were classified as previously exposed to *P. falciparum* or if the current *P. falciparum* infection was their first exposure. Infection with *P. falciparum* parasites is known to induce a robust IgG response in humans with IgG_1_ levels showing the highest titers followed by IgG_3_, IgG_4_, and IgG_2_ (6–9). Multiple individual *P. falciparum* antigens have been identified as immunogens in the human host and utilized in candidate malaria vaccine development, association with clinical disease, or seroepidemiological studies (15, 16, 26, 38, 39). Previous work has estimated that upon natural exposure to *P. falciparum* and generation of IgG_1_ antibodies, children would remain seropositive to this subclass for an estimated 408 days for PfMSP1 and 153 days for PfAMA1 (5). Studies by others evaluating all age ranges (likely from persons with multiple past infections) have also confirmed this longevity of IgG to these two antigens with estimates for IgG half-life in the host of years to decades (40, 41). Ultimately, quantitative empirical estimates for an individual’s retention of IgG antibodies against any *P. falciparum* antigen would be a factor of numerous immunological and parasitological factors, so broad assumptions could not be made for a human population. Interestingly, though known to both be among the longer-lasting anti-*Plasmodium* antibodies, IgG levels to the PfMSP1 and PfAMA1 antigens show low correlation in individuals (42), and it has been hypothesized that different factors control antibody responses to these antigens (43). Based on this information from previous studies, binary classification for this study population to estimate *P. falciparum* exposure appeared to be appropriate based on IgG_1_ responses to both of these antigens. To reduce classification error, an additional margin was added to the binary categorization, so only those children with much higher IgG_1_ levels (well beyond the seropositivity threshold) were considered as non-naïve for *P. falciparum*. This classification scheme is also supported by the three TES enrollment sites being located in meso- to high-endemic *P. falciparum* settings in Angola (34), and the relatively young ages of participants (6 months to 11 years old), meaning it is unlikely they would have had enough years of life for IgG_1_ seroreversion from a previous *P. falciparum* exposure. A previous report has also shown rapid acquisition of total IgG against *P. falciparum* antigens with time spent in Angola (44), indicating the high endemic nature of *P. falciparum* in this setting.

Between these two classification groups, no significant difference was noted between the peripheral parasite densities at presentation to the health facility. It may be expected that previous malaria exposure would suppress parasite burden (1, 32), but all children enrolled in this study were symptomatic, so the distributions presented here are not inclusive of lower-density asymptomatic infections in the general population (45, 46). Classification of the study population into naïve and non-naïve categories found many differences that highlighted classical assumptions of human adaptive humoral immunity. Additionally, as only children older than 6 months old were included in this study, any maternal antibodies would have likely been eliminated by this time (18, 19), and this Ig data most certainly represents true host response. The current *P. falciparum* infection for non-naïve individuals appeared to have served as an antibody boosting event for IgG_1_, IgG_3_, IgM, and IgA antibodies when compared to naïve persons, with maximum antibody levels (C_max_) significantly higher for all four of these Igs, though IgM boosting appears to be the most subdued when compared to the other isotypes/subclasses. Similar to IgG boosting seen in humans after successive *P. falciparum* infections (37, 47), IgG_1_ antibodies showed the most antigen targets with C_max_ values significantly higher (11/32, 34.4% of all antigens) for non-naïve vs. naïve children, though IgG_3_ and IgA also showed multiple antigen targets (9 each) exhibiting boosting characteristics. Specifically, the IgG_1_ responses to Rh2030, HSP40, and PfAMA1 were the highest boosted levels for non-naïve participants, and may (collectively or individually) be used to predict previous or nascent exposure. A prior study showed that in previously exposed individuals, the Rh2030 and PfAMA1 responses were highly correlated with each other and predictive of the *P. falciparum* pre-patent period (32). Recently, IgG sero-responses to all three of these antigens were significantly correlated to asymptomatic infection, whereas responses to the Etramp5Ag1 and PfMSP1 antigen were correlated with clinical disease (48), giving evidence that the non-naïve children in this current study had some form of previous *P. falciparum* exposure and were boosted during the current infection.

Recent work has expanded on contribution of the IgM response to *P. falciparum* infection with findings of IgM-positive memory B cell subsets being predominant in children, IgM inhibiting parasite invasion in a complement-dependent manner, and persistence of IgM response to merozoite surface antigen over time (26, 27). Among all four Ig isotypes/subclasses tested here, estimates for change in day of enrollment to peak Ig levels (Δ_C_) were universally higher for IgM response in the naïve individuals, with 23/32 (71.9%) *P. falciparum* antigen Δ_C_ responses reaching statistical significance. These data suggest the presence of IgM + memory B cell response in non-naïve children (49), as the IgM response in naïve children is practically non-existent at the day of enrollment and 11/32 (34.4%) of IgM t_max_ estimates higher in naïves. Induction of IgA during natural (3, 50) and malaria vaccine (51) exposure has been well documented, though it’s unclear if there’s a specific immunological role this isotype plays in response to *P. falciparum* infection. Previous work to assess the value of IgA in protecting the host against malaria pathogenesis found no significant benefit from anti-PfMSP1 IgA when infecting mice with transgenic *P. berghei* expressing the PfMSP1 antigen (52). In the same manner as IgM, Δ_C_ estimates for IgA were nearly all higher in naïve children, but more similar to IgG_1_ and IgG_3_, C_max_ of nearly all antigens were higher in non-naïves. This data shows evidence for IgA boosting upon re-exposure similar to IgG subclasses with PfAMA1, Rh_2030, and HSP40 boosting as providing some of the strongest markers for previous exposure.

A limitation of this study was that presence and magnitude of IgG_1_ response against two *P. falciparum* antigens were used as the only proxy for classification of any previous exposure. However, although previous studies from endemic settings have followed up persons and assessed Ig dynamics over long periods of time (8), previous malaria history (if assessed) comes from clinical episodes, and would miss asymptomatic infections. Additionally, infection events from longitudinal studies are typically noted by sparse intervals or clinical episodes, so true *P. falciparum* exposure could be missed by the sampling design. This current study only measures the absolute level of antibodies binding to specific *P. falciparum* antigens, and experiments were not performed to measure binding strength among different antigens or the naïve/non-naïve groups. Proportions of B cell subsets were not able to be evaluated, nor was antibody functional activity (inhibition of parasite invasion, complement activation, etc.) assessed. The results presented here are data only from symptomatic Angolan children infected with *P. falciparum* from Angola, so it is possible that persons of older ages, persons living in different transmission settings, or different host and parasite genotypes would provide different outputs than the ones observed here. More robust statistical methods for looking across data from multiple Ig classes and antigens are also needed to gain a comprehensive understanding of the human B cell response to *P. falciparum* infection.

Classification of children into *P. falciparum* naïve and non-naïve categories and assessment of antibody dynamics weeks after resolved infection showed stark differences in Ig levels and temporal trends between these two groups. This data helps to elucidate Ig dynamics in a human population naturally exposed to *P. falciparum* malaria and provides generalizable results which can better assist in translating findings from seroepidemiological studies. Presentation here of Ig results by individual *P. falciparum* antigens will also aid future research studies utilizing these specific targets to put serological data into context

## Data availability statement

The original contributions presented in this study are included in the article/Supplementary Material, further inquiries can be directed to the corresponding author.

## Author contributions

PD and MP designed and coordinated the field study. ER and MP designed the laboratory study, conceptualized the experiments, drafted the manuscript, and performed statistical analyses. ER and DN performed laboratory assays. BW, JB, CD, and KT provided antigens and scientific expertise. All authors reviewed and approved the final version of the manuscript.

## Conflict of interest

The authors declare that the research was conducted in the absence of any commercial or financial relationships that could be construed as a potential conflict of interest.

## Publisher’s note

All claims expressed in this article are solely those of the authors and do not necessarily represent those of their affiliated organizations, or those of the publisher, the editors and the reviewers. Any product that may be evaluated in this article, or claim that may be made by its manufacturer, is not guaranteed or endorsed by the publisher.
