# Supplementary material for: Antibody dynamics in children with first or repeat Plasmodium falciparum infections
Source: Front Med (Lausanne). 2022 Jul 19;9:869028. doi: 10.3389/fmed.2022.869028 (PMC9343764; doi:10.3389/fmed.2022.869028)
Supplement: Supplementary file 1 [file Data_Sheet_1.pdf]

Supplementary Material

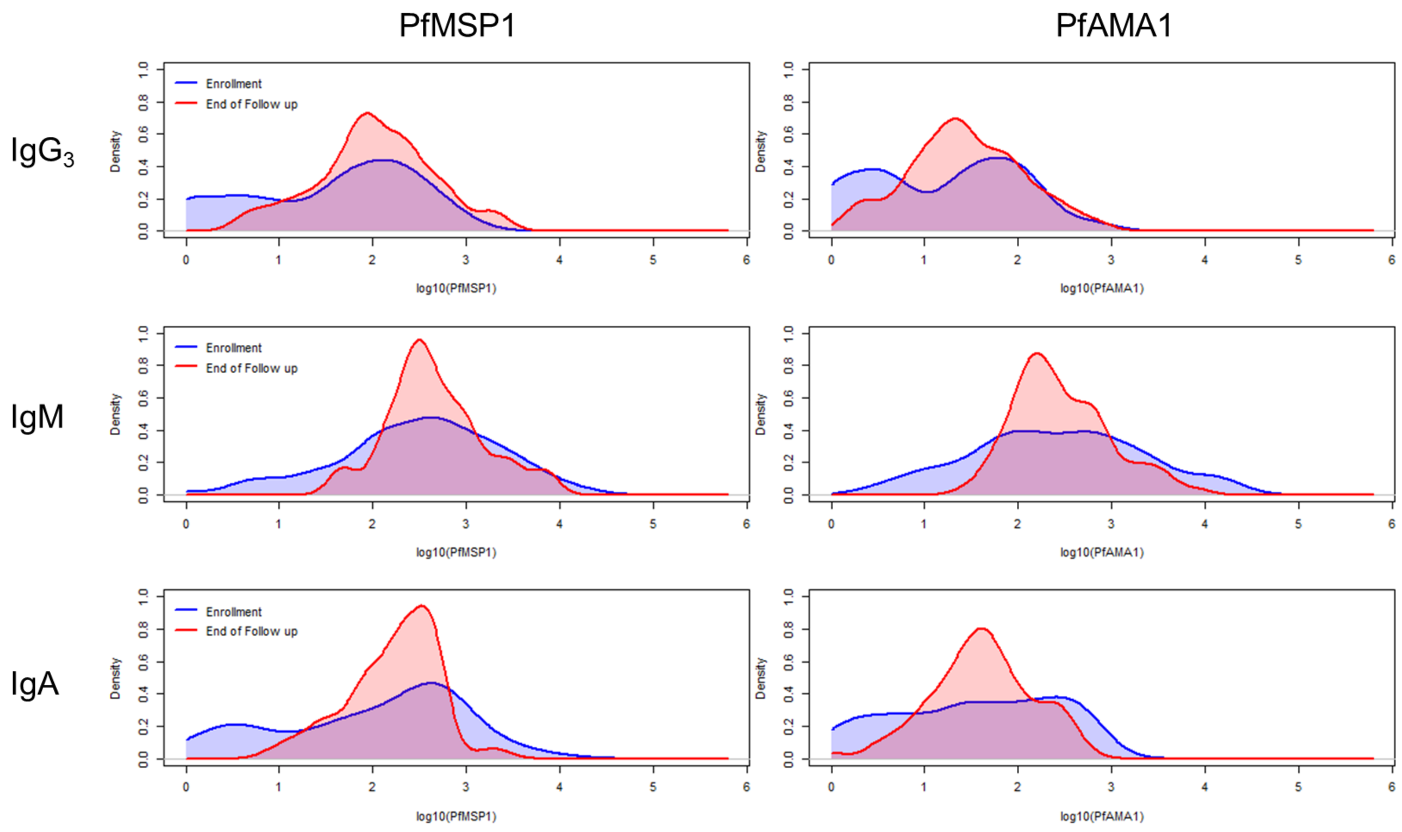

Supplementary Figure 1. Smoothed distribution of IgG_3, IgM, and IgA, antibody responses to PfMSP1 (left) and PfAMA1 (right) at enrollment at baseline and last day of follow up in children treated for *P. falciparum* infection. Distribution of Ig levels at day of enrollment (presentation at health facility) shown in blue and last day of follow-up in red. Assay signals displayed on x-axes as log_{10} transformed.

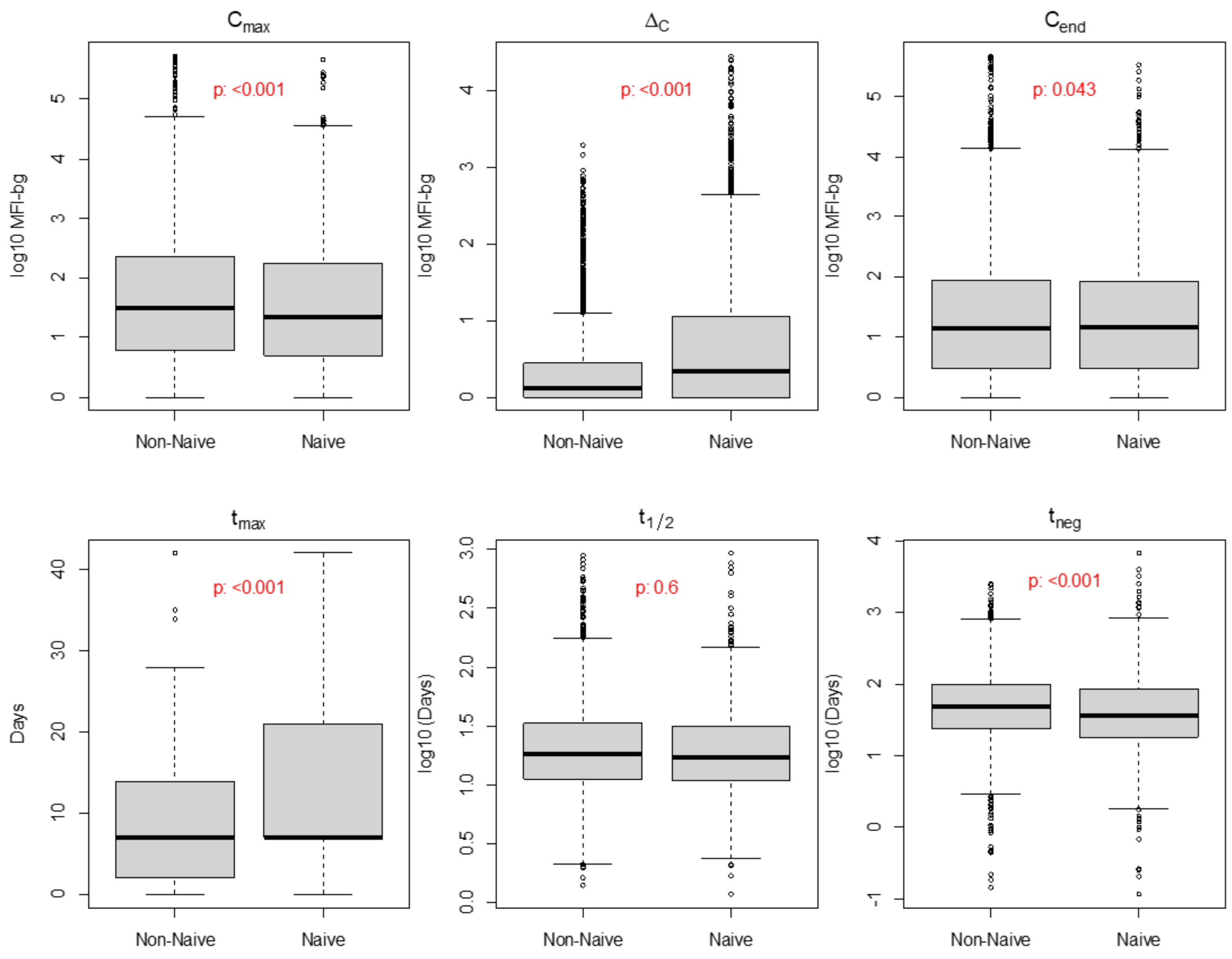

Supplementary Figure 2. Distribution of key post-treatment clearance parameters in non-naïve and naïve Angolan children treated for malaria, aggregated for all immunoglobulin classes and subclasses. P-value for Kolmogorov-Smirnov test for difference in empiric distribution between non-naïve and naïve participants.

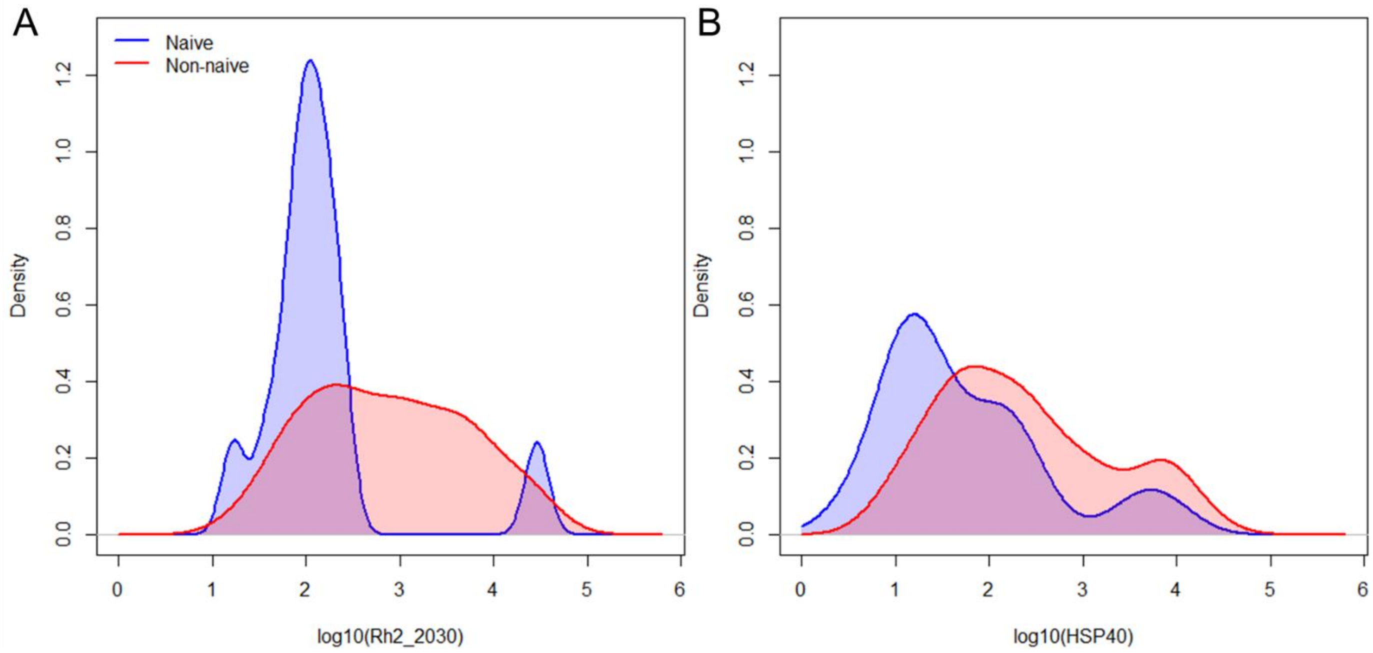

Supplementary Figure 3. Histograms of the distribution of IgG_1 antibody responses. To Rh2030 (A) and HSP40 (B) on the last day of follow up in children treated for *P. falciparum* infection, stratifying by likely naïve or non-naïve classification.
